# Synthesis and crystal structure of a new copper(II) complex based on 5-ethyl-3-(pyridin-2-yl)-1,2,4-triazole

**DOI:** 10.1107/S2056989023003079

**Published:** 2023-04-14

**Authors:** Yuliia P. Petrenko, Dmytro M. Khomenko, Roman O. Doroshchuk, Ilona V. Raspertova, Sergiu Shova, Rostyslav D. Lampeka

**Affiliations:** a Department of Chemistry, Taras Shevchenko National University of Kyiv, Volodymyrska str. 64/13, 01601 Kyiv, Ukraine; b Enamine Ltd., Chervonotkatska Street 78, Kyiv 02094, Ukraine; c "PetruPoni" Institute of Macromolecular Chemistry, Aleea Gr., GhicaVoda 41A, 700487 Iasi, Romania; Texas A & M University, USA

**Keywords:** copper(II) complex, X-ray crystallography, acetate anion, 3-(2-pyrid­yl)-1,2,4-triazole, crystal packing

## Abstract

The title compound, bis­[μ-3-ethyl-5-(pyridin-2-yl)-1*H*-1,2,4-triazol-1-ido]bis[acetato­(di­methyl­formamide)­copper(II)], [Cu_2_(C_9_H_9_N_4_)_2_(C_2_H_3_O_2_)_2_(C_3_H_7_NO)_2_] or [Cu_2_(*L* ^Et^)_2_(OAc)_2_(dmf)_2_], is a triazolate complex, which contains two 3-(2-pyrid­yl)-5-ethyl-triazolates (*L* ^Et^)^−^ in bidentate-bridged coordination modes. Both copper atoms are involved in the formation of a planar six-membered metallocycle Cu–[N—N]_2_–Cu. The inversion center of the complex is located at the mid-point of the Cu⋯Cu vector. Each Cu^II^ atom has a distorted trigonal–bipyramidal environment formed by the three nitro­gen atoms of the deprotonated bridging 3-(2-pyrid­yl)-5-ethyl-triazolate unit, oxygen atoms of the OAc^−^ group and dmf mol­ecule. In the crystal, C—H⋯O hydrogen bonds link the mol­ecules into chains running along the *c*-axis direction.

## Abstract 2

A new copper(II) coordination compound [Cu_2_(*L* ^Et^)_2_(OAc)_2_(dmf)_2_], where H*L* ^Et^ = 3-(2-pyrid­yl)-5-ethyl-1,2,4-triazole, was synthesized and structurally characterized by single-crystal X-ray diffraction.

## 1. Chemical context

The design and construction of coordination complexes based on dinuclear copper(II) compounds have been the subject of intensive study over the past decades (Li *et al.*, 2018 ▸; Cui *et al.*, 2019 ▸; Doroschuk, 2016 ▸). N-containing ligands with polypyridyl (Lee *et al.*, 2017 ▸), triazolyl (Kucheriv *et al.*, 2016 ▸) and pyridyl moieties (Bartual-Murgui *et al.*, 2020 ▸) have been widely used for this purpose. Much inter­est has been focused on functional materials with the presence of a triazole ring, which demonstrate inter­esting properties such as catalytic ability (Petrenko *et al.*, 2021 ▸), anti­cancer activity (Muhammad & Guo, 2014 ▸) and magnetism (Kuzevanova *et al.*, 2021 ▸). Although a variety of triazolate frameworks with intriguing topologies (Govor *et al.*, 2010 ▸; Senchyk *et al.*, 2012 ▸; Lysenko *et al.*, 2016 ▸) have been synthesized to date, making rational control in the construction of coordination compounds is a great challenge in crystal engineering. Derivatives of 3-(2-pyrid­yl)-1,2,4-triazole are among the most widely used ligands that form stable Cu^II^ coordination compounds. There are about 127 examples in the Cambridge Structural Database that exhibit this type of ligand, 37 of which complexes include the binuclear unit [Cu_2_(trz-py)_2_] with a Cu–[N—N]_2_–Cu bridge. Among the reported binuclear compounds, there are few reports of 3-(2-pyrid­yl)-1,2,4-triazole compounds obtained with copper(II) acetate (Petrenko *et al.*, 2021 ▸; Li *et al.*, 2010 ▸). In all cases, the equatorial coordination consists of metallocentres linked by two deprotonated triazole ligands, where additional ligands (acetate anions or solvent) axially coordinate the copper atom. 
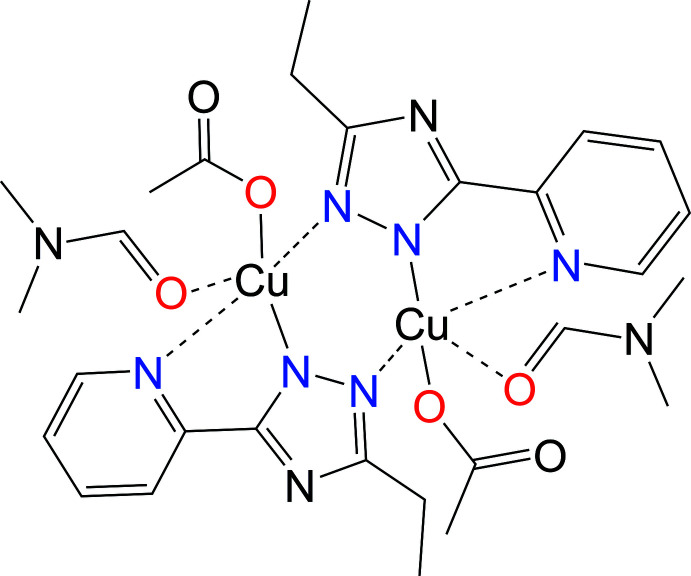


## 2. Structural commentary

**Figure 1:**
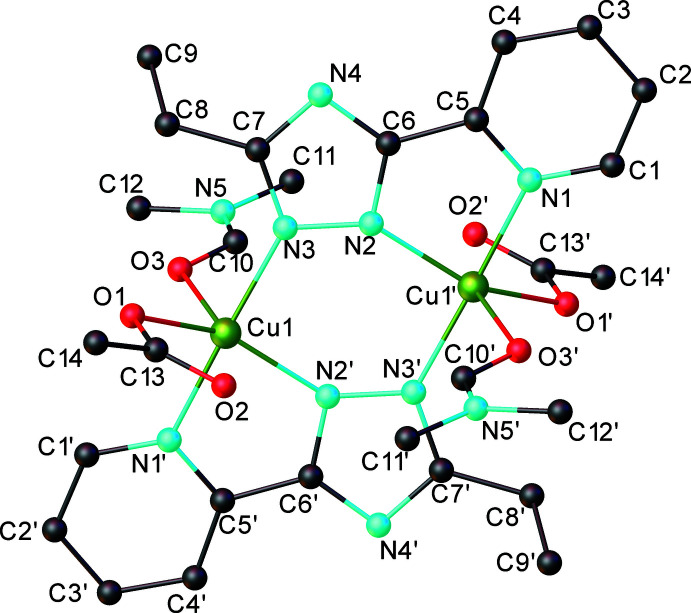
X-ray mol­ecular structure with atom labelling for [Cu_2_(*L* ^Et^)_2_(OAc)_2_(dmf)_2_].

The results of the X-ray diffraction study are depicted in Fig. 1 ▸. The crystal is built from discrete dinuclear units [Cu_2_(*L* ^Et^)_2_(OAc)_2_(dmf)_2_], where the Cu⋯Cu1′ separation is of 4.0159 (8) Å. There are no co-crystallized solvent mol­ecules in the crystals. The complex mol­ecule has its own crystallographically imposed symmetry, being assembled around the inversion centers located at the mid-point of the Cu1⋯Cu1′ distances. Each copper(II) atom exhibits an N_3_O_2_ coordination environment in a slightly distorted trigonal–bipyramidal geometry provided by three nitro­gen atoms of the organic ligands and two oxygen atoms from the dmf mol­ecule and the monodentate acetate anion.

**Figure 2:**
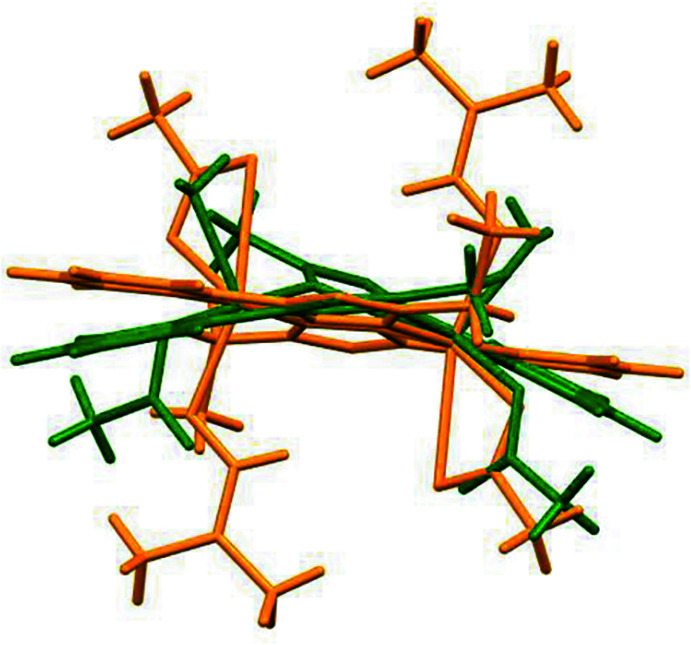
Overlay diagram of the mol­ecular structures [Cu_2_(*L* ^Me^)_2_(OAc)_2_(H_2_O)_2_] (green) and [Cu_2_(*L* ^Et^)_2_(OAc)_2_(dmf)_2_] (yellow), showing the difference in the spatial arrangement of the ligands.

The inner (Cu1/N2/N3–Cu1′/N2′/N3′) core has an almost planar conformation in [Cu_2_(*L* ^Et^)_2_(OAc)_2_(dmf)_2_], although for the previously described complex [Cu_2_(*L* ^Me^)_2_(OAc)_2_(H_2_O)_2_] [H*L* ^Me^ = 5-methyl-3-(2-pyrid­yl)-1,2,4-triazole], a twist–boat conformation was observed (Petrenko *et al.*, 2021 ▸) for the non-planar six-membered Cu_2_N_4_ metal ring. The structures were compared (Fig. 2 ▸) using *OLEX2* software (Dolomanov *et al.*, 2009 ▸). It was found that in [Cu_2_(*L* ^Me^)_2_(OAc)_2_(H_2_O)_2_], the water mol­ecules are axially coordinated by the central atom from one side of the Cu_2_N_4_ plane, and the acetates from the other. Thus, the non-coordinated oxygen of the acetate anion is involved in an inter­molecular hydrogen bond with the coordinated water mol­ecule of an adjacent complex, giving rise to an essentially different crystal motif than was observed for [Cu_2_(*L* ^Et^)_2_(OAc)_2_(dmf)_2_]. In the newly reported compound [Cu_2_(*L* ^Et^)_2_(OAc)_2_(dmf)_2_], the copper atoms coordinate the dmf mol­ecules and acetate anions in the axial positions in such a manner that they reflect in the symmetry center, which is typical for such a kind of binuclear species. Notably, both [Cu_2_(*L* ^Me^)_2_(OAc)_2_(H_2_O)_2_] and [Cu_2_(*L* ^Et^)_2_(OAc)_2_(dmf)_2_] were synthesized using the same conditions. These features can be probably induced by different substituents in the 5-position of the 3-(2-pyrid­yl)-1,2,4-triazole ring in these two compounds, indicating that even negligible changes of the non-coordinating part of the ligand could significantly influence the structure of the complex. The non-typical mol­ecular structure of [Cu_2_(*L* ^Me^)_2_(OAc)_2_(H_2_O)_2_] is supported by the formation of inter­molecular hydrogen bonds. In the case of [Cu_2_(*L* ^Et^)_2_(OAc)_2_(dmf)_2_], branching of the non-coordinated part leads to the formation of a less-hindered structure of higher symmetry, similar to those of the previously described 37 compounds, indicating a small difference in the energies of these two topologies, which is probably the result of the formation of additional inter­molecular contacts.

## 3. Supra­molecular features

**Figure 3:**
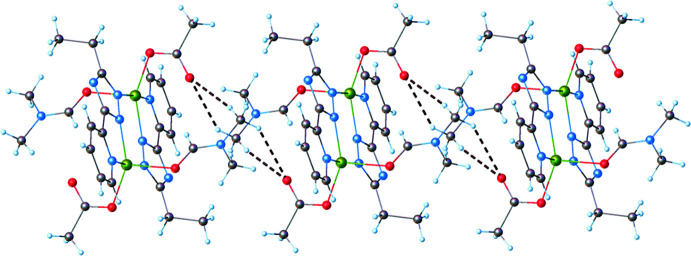
One-dimensional coordination network viewed along the *b*-axis.

**Figure 4:**
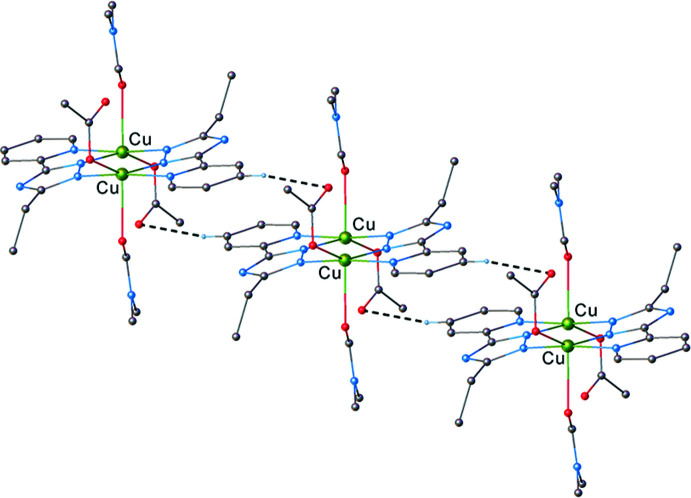
Partial view of the crystal packing showing hydrogen-bond contacts between adjacent mol­ecules.

Further analysis of the structure showed that the crystal structure motif is characterized as a parallel packing of discrete supra­molecular chains running along the *b*-axis direction (Fig. 3 ▸). Within a chain, the complex mol­ecules inter­act through weak C—H⋯O hydrogen bonds, where the pyridine H atom acts as acceptor, and the acetate O atom as donor (Table 1 ▸, Fig. 4 ▸).

## 4. Database survey

A search of the Cambridge Structural Database (CSD version 5.43, update of March 2022; Groom *et al.*, 2016 ▸) using *ConQuest* (Bruno *et al.*, 2002 ▸) revealed 127 hits for the moiety containing the Cu_2_(trz-py)_2_ unit. In addition, the searches were also limited to structures with low *R*-factor values (*R* < 0.05). Most similar to the title compound are binuclear copper(II) complexes with two unsubstituted 3-(2-pyrid­yl)-1,2,4-triazole ligands, two anions and two water mol­ecules in the axial positions [DODRIX, DODRET (Prins *et al.*, 1985 ▸); FIVGEY (Matthews *et al.*, 2003 ▸)] and with 3,5-bis­(2-pyrid­yl)-1,2,4-triazole ligands (JUDBIV; Du *et al.*, 2017 ▸). The compounds most closely related to the title complex are binuclear Cu^II^ complexes with unsubstituted 3-(2-pyrid­yl)-1,2,4-triazole ligands and a coordinated acetate anion [UQEQUD (Li *et al.*, 2011 ▸); GUWZEE (Li *et al.*, 2010 ▸); CUSHUV (Li *et al.*, 2015 ▸) and JUDBOB (Du *et al.*, 2017 ▸)].

## 5. Synthesis and crystallization

Ligand H*L* ^Et^ was prepared according to the synthesis described in the literature (Khomenko *et al.*, 2016 ▸; Zakharchenko *et al.*, 2019 ▸). Single crystals of [Cu_2_(*L* ^Et^)2(OAc)_2_(dmf)_2_] were obtained in dmf. A solution of Cu(OAc)_2_·H_2_O (0.50 g, 10 ml, 2.5 mmol) was added to a solution of H*L* ^Et^ (0.48 g, 5 ml, 2.5 mmol). The resulting mixture was stirred with heating for 15 min, and then left in the air for crystallization. The green crystals obtained were filtered off, washed with dmf and dried in air. Yield 0.507 g (55%). Analysis calculated for C_28_H_38_Cu_2_N_10_O_6_ (%): C 45.58, H 5.19, N 18.99; found: C 45.57, H 5.17, N 18.96.

## 6. Refinement

Crystal data, data collection and structure refinement details are summarized in Table 2 ▸.

## Supplementary Material

Crystal structure: contains datablock(s) I. DOI: 10.1107/S2056989023003079/jy2029sup1.cif

Structure factors: contains datablock(s) I. DOI: 10.1107/S2056989023003079/jy2029Isup2.hkl

CCDC reference: 2253664

Additional supporting information:  crystallographic information; 3D view; checkCIF report

## Figures and Tables

**Table 1:** Hydrogen-bond geometry (Å, °)

*D*—H⋯*A*	*D*—H	H⋯*A*	*D*⋯*A*	*D*—H⋯*A*
C3—H3⋯O2^i^	0.93	2.59	3.513 (4)	170

Symmetry code: (i) 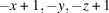 .

**Table 2:** Experimental details

Crystal data	
Chemical formula	[Cu_2_(C_9_H_9_N_4_)_2_(C_2_H_3_O_2_)_2_(C_3_H_7_NO)_2_]
*M*_r_	737.76
Crystal system, space group	Monoclinic, *P*2_1_/*c*
Temperature (K)	293
*a*, *b*, *c* (Å)	9.4445 (5), 8.9404 (4), 20.2237 (9)
β (°)	93.257 (4)
*V* (Å^3^)	1704.88 (14)
*Z*	2
Radiation type	Mo *K*α
μ (mm^−1^)	1.30
Crystal size (mm)	0.3 × 0.2 × 0.15

Data collection	
Diffractometer	Xcalibur, Eos
Absorption correction	Multi-scan (*CrysAlis PRO*; Rigaku OD, 2019 ▸)
*T*_min_, *T*_max_	0.876, 1.000
No. of measured, independent and observed [*I* > 2σ(*I*)] reflections	7405, 3007, 2382
*R*_int_	0.034
(sin θ/λ)_max_ (Å^−1^)	0.595

Refinement	
*R*[*F* ^2^ > 2σ(*F* ^2^)], *wR*(*F* ^2^), *S*	0.039, 0.085, 1.05
No. of reflections	3007
No. of parameters	212
H-atom treatment	H-atom parameters constrained
Δρ_max_, Δρ_min_ (e Å^−3^)	0.31, −0.27

Computer programs: *CrysAlis PRO* (Rigaku OD, 2019 ▸), *SHELXT* (Sheldrick, 2015*a* ▸), *SHELXL2018/3* (Sheldrick, 2015*b* ▸) and *OLEX2* (Dolomanov *et al.*, 2009 ▸).

## supplementary crystallographic information

### Crystal data

**Table:**

[Cu_2_(C_9_H_9_N_4_)_2_(C_2_H_3_O_2_)_2_(C_3_H_7_NO)_2_]	*F*(000) = 764
*M**_r_* = 737.76	*D*_x_ = 1.437 Mg m^−^^3^
Monoclinic, *P*2_1_/*c*	Mo *K*α radiation, λ = 0.71073 Å
*a* = 9.4445 (5) Å	Cell parameters from 2231 reflections
*b* = 8.9404 (4) Å	θ = 2.0–25.3°
*c* = 20.2237 (9) Å	µ = 1.30 mm^−^^1^
β = 93.257 (4)°	*T* = 293 K
*V* = 1704.88 (14) Å^3^	Prism, clear dark blue
*Z* = 2	0.3 × 0.2 × 0.15 mm

### Data collection

**Table:**

Xcalibur, Eos diffractometer	3007 independent reflections
Radiation source: fine-focus sealed X-ray tube, Enhance (Mo) X-ray Source	2382 reflections with *I* > 2σ(*I*)
Graphite monochromator	*R*_int_ = 0.034
Detector resolution: 16.1593 pixels mm^-1^	θ_max_ = 25.0°, θ_min_ = 2.0°
ω scans	*h* = −11→10
Absorption correction: multi-scan (CrysAlisPro; Rigaku OD, 2019)	*k* = −8→10
*T*_min_ = 0.876, *T*_max_ = 1.000	*l* = −18→24
7405 measured reflections	

### Refinement

**Table:**

Refinement on *F*^2^	Primary atom site location: dual
Least-squares matrix: full	Hydrogen site location: inferred from neighbouring sites
*R*[*F*^2^ > 2σ(*F*^2^)] = 0.039	H-atom parameters constrained
*wR*(*F*^2^) = 0.085	*w* = 1/[σ^2^(*F*_o_^2^) + (0.0304*P*)^2^ + 0.6113*P*] where *P* = (*F*_o_^2^ + 2*F*_c_^2^)/3
*S* = 1.05	(Δ/σ)_max_ < 0.001
3007 reflections	Δρ_max_ = 0.31 e Å^−^^3^
212 parameters	Δρ_min_ = −0.27 e Å^−^^3^
0 restraints	

### Special details

**Table:**

Geometry. All e.s.d.'s (except the e.s.d. in the dihedral angle between two l.s. planes) are estimated using the full covariance matrix. The cell e.s.d.'s are taken into account individually in the estimation of e.s.d.'s in distances, angles and torsion angles; correlations between e.s.d.'s in cell parameters are only used when they are defined by crystal symmetry. An approximate (isotropic) treatment of cell e.s.d.'s is used for estimating e.s.d.'s involving l.s. planes.

### Fractional atomic coordinates and isotropic or equivalent isotropic displacement parameters (Å^2^)

**Table:**

	*x*	*y*	*z*	*U*_iso_*/*U*_eq_	
Cu1	0.48907 (4)	0.60148 (4)	0.58814 (2)	0.03442 (13)	
O1	0.4294 (2)	0.5477 (2)	0.67766 (10)	0.0415 (5)	
O2	0.2385 (3)	0.4889 (3)	0.61484 (12)	0.0601 (7)	
O3	0.7170 (2)	0.6884 (3)	0.60652 (12)	0.0528 (6)	
N1	0.5774 (3)	0.1842 (3)	0.39823 (12)	0.0353 (6)	
N2	0.5539 (2)	0.3380 (3)	0.50737 (11)	0.0327 (6)	
N3	0.5690 (2)	0.4020 (2)	0.56906 (11)	0.0327 (6)	
N4	0.6816 (3)	0.1801 (3)	0.57376 (12)	0.0404 (6)	
N5	0.9247 (3)	0.7291 (3)	0.55819 (14)	0.0521 (7)	
C1	0.5741 (3)	0.1078 (4)	0.34135 (16)	0.0457 (8)	
H1	0.537481	0.154425	0.302982	0.055*	
C2	0.6226 (4)	−0.0369 (4)	0.33712 (17)	0.0544 (9)	
H2	0.618426	−0.086813	0.296711	0.065*	
C3	0.6776 (4)	−0.1069 (4)	0.39395 (19)	0.0551 (10)	
H3	0.712383	−0.204028	0.392220	0.066*	
C4	0.6800 (3)	−0.0302 (3)	0.45327 (17)	0.0461 (8)	
H4	0.715879	−0.075095	0.492185	0.055*	
C5	0.6281 (3)	0.1149 (3)	0.45383 (15)	0.0355 (7)	
C6	0.6231 (3)	0.2074 (3)	0.51308 (14)	0.0337 (7)	
C7	0.6464 (3)	0.3047 (3)	0.60695 (15)	0.0376 (7)	
C8	0.6944 (4)	0.3322 (4)	0.67766 (16)	0.0512 (9)	
H8A	0.656363	0.254626	0.705100	0.061*	
H8B	0.656717	0.427334	0.691653	0.061*	
C9	0.8546 (4)	0.3344 (5)	0.6883 (2)	0.0881 (14)	
H9A	0.892006	0.418177	0.665222	0.132*	
H9B	0.892983	0.243409	0.671529	0.132*	
H9C	0.880205	0.342977	0.734718	0.132*	
C10	0.7957 (3)	0.6721 (3)	0.56038 (17)	0.0453 (8)	
H10	0.761756	0.615416	0.524331	0.054*	
C11	1.0110 (4)	0.7075 (4)	0.5014 (2)	0.0706 (12)	
H11A	1.096094	0.654624	0.515113	0.106*	
H11B	1.035153	0.803030	0.483543	0.106*	
H11C	0.958395	0.650492	0.468059	0.106*	
C12	0.9843 (5)	0.8163 (6)	0.6132 (2)	0.0978 (16)	
H12A	0.975052	0.920858	0.603018	0.147*	
H12B	1.082830	0.791748	0.620908	0.147*	
H12C	0.934694	0.794217	0.652151	0.147*	
C13	0.3033 (4)	0.4974 (3)	0.66937 (16)	0.0407 (8)	
C14	0.2343 (4)	0.4436 (4)	0.73102 (18)	0.0660 (11)	
H14A	0.221006	0.337203	0.728592	0.099*	
H14B	0.144052	0.491720	0.733999	0.099*	
H14C	0.294255	0.467671	0.769470	0.099*	

### Atomic displacement parameters (Å^2^)

**Table:**

	*U*^11^	*U*^22^	*U*^33^	*U*^12^	*U*^13^	*U*^23^
Cu1	0.0376 (2)	0.0393 (2)	0.0265 (2)	0.00146 (18)	0.00264 (15)	0.00220 (17)
O1	0.0415 (14)	0.0516 (13)	0.0316 (12)	−0.0047 (11)	0.0043 (10)	−0.0001 (10)
O2	0.0539 (16)	0.0787 (17)	0.0469 (15)	−0.0069 (13)	−0.0063 (12)	−0.0004 (13)
O3	0.0386 (14)	0.0636 (15)	0.0570 (16)	−0.0071 (11)	0.0085 (12)	−0.0052 (12)
N1	0.0375 (15)	0.0373 (14)	0.0314 (15)	−0.0007 (12)	0.0051 (11)	−0.0003 (12)
N2	0.0348 (15)	0.0356 (13)	0.0276 (14)	0.0014 (11)	0.0016 (11)	0.0052 (11)
N3	0.0357 (15)	0.0376 (13)	0.0247 (13)	0.0019 (12)	0.0009 (11)	0.0038 (11)
N4	0.0430 (16)	0.0435 (15)	0.0345 (15)	0.0071 (13)	0.0000 (12)	0.0061 (13)
N5	0.0349 (17)	0.0643 (18)	0.057 (2)	−0.0065 (14)	0.0026 (14)	0.0023 (15)
C1	0.051 (2)	0.049 (2)	0.0379 (19)	−0.0010 (16)	0.0040 (16)	−0.0029 (16)
C2	0.071 (3)	0.048 (2)	0.045 (2)	−0.0043 (19)	0.0099 (19)	−0.0101 (18)
C3	0.063 (3)	0.0392 (18)	0.064 (3)	0.0001 (17)	0.010 (2)	−0.0086 (18)
C4	0.048 (2)	0.0411 (18)	0.049 (2)	0.0041 (16)	0.0020 (16)	0.0061 (16)
C5	0.0305 (17)	0.0371 (17)	0.0396 (18)	−0.0026 (14)	0.0069 (13)	0.0016 (14)
C6	0.0298 (17)	0.0393 (17)	0.0322 (17)	0.0000 (14)	0.0046 (13)	0.0061 (14)
C7	0.0348 (18)	0.0452 (18)	0.0327 (18)	−0.0012 (15)	0.0003 (14)	0.0082 (15)
C8	0.059 (2)	0.059 (2)	0.0346 (19)	0.0106 (18)	−0.0078 (16)	0.0043 (17)
C9	0.072 (3)	0.133 (4)	0.056 (3)	−0.007 (3)	−0.025 (2)	0.010 (3)
C10	0.037 (2)	0.0445 (19)	0.054 (2)	−0.0045 (16)	−0.0035 (16)	0.0055 (17)
C11	0.050 (2)	0.088 (3)	0.076 (3)	−0.006 (2)	0.016 (2)	0.006 (2)
C12	0.065 (3)	0.135 (4)	0.094 (4)	−0.042 (3)	0.009 (3)	−0.035 (3)
C13	0.046 (2)	0.0380 (18)	0.038 (2)	0.0015 (16)	0.0051 (16)	0.0002 (14)
C14	0.064 (3)	0.082 (3)	0.054 (2)	−0.018 (2)	0.023 (2)	0.005 (2)

### Geometric parameters (Å, º)

**Table:**

Cu1—O1	1.985 (2)	C3—C4	1.381 (4)
Cu1—O3	2.299 (2)	C4—H4	0.9300
Cu1—N1^i^	2.040 (2)	C4—C5	1.387 (4)
Cu1—N2^i^	2.025 (2)	C5—C6	1.459 (4)
Cu1—N3	1.983 (2)	C7—C8	1.496 (4)
O1—C13	1.276 (4)	C8—H8A	0.9700
O2—C13	1.233 (4)	C8—H8B	0.9700
O3—C10	1.234 (4)	C8—C9	1.516 (5)
N1—C1	1.337 (4)	C9—H9A	0.9600
N1—C5	1.348 (4)	C9—H9B	0.9600
N2—N3	1.373 (3)	C9—H9C	0.9600
N2—C6	1.340 (3)	C10—H10	0.9300
N3—C7	1.347 (3)	C11—H11A	0.9600
N4—C6	1.339 (3)	C11—H11B	0.9600
N4—C7	1.351 (4)	C11—H11C	0.9600
N5—C10	1.323 (4)	C12—H12A	0.9600
N5—C11	1.459 (4)	C12—H12B	0.9600
N5—C12	1.446 (5)	C12—H12C	0.9600
C1—H1	0.9300	C13—C14	1.518 (4)
C1—C2	1.377 (4)	C14—H14A	0.9600
C2—H2	0.9300	C14—H14B	0.9600
C2—C3	1.384 (5)	C14—H14C	0.9600
C3—H3	0.9300		
			
O1—Cu1—O3	104.24 (9)	N4—C6—N2	114.3 (3)
O1—Cu1—N1^i^	89.93 (9)	N4—C6—C5	128.2 (3)
O1—Cu1—N2^i^	151.98 (9)	N3—C7—N4	113.0 (3)
N1^i^—Cu1—O3	87.31 (9)	N3—C7—C8	124.2 (3)
N2^i^—Cu1—O3	101.48 (9)	N4—C7—C8	122.7 (3)
N2^i^—Cu1—N1^i^	80.29 (10)	C7—C8—H8A	109.1
N3—Cu1—O1	95.20 (9)	C7—C8—H8B	109.1
N3—Cu1—O3	88.40 (9)	C7—C8—C9	112.5 (3)
N3—Cu1—N1^i^	173.99 (10)	H8A—C8—H8B	107.8
N3—Cu1—N2^i^	96.46 (9)	C9—C8—H8A	109.1
C13—O1—Cu1	106.07 (19)	C9—C8—H8B	109.1
C10—O3—Cu1	115.9 (2)	C8—C9—H9A	109.5
C1—N1—Cu1^i^	127.1 (2)	C8—C9—H9B	109.5
C1—N1—C5	118.2 (3)	C8—C9—H9C	109.5
C5—N1—Cu1^i^	114.60 (19)	H9A—C9—H9B	109.5
N3—N2—Cu1^i^	139.50 (18)	H9A—C9—H9C	109.5
C6—N2—Cu1^i^	112.67 (19)	H9B—C9—H9C	109.5
C6—N2—N3	105.1 (2)	O3—C10—N5	125.1 (3)
N2—N3—Cu1	122.07 (17)	O3—C10—H10	117.4
C7—N3—Cu1	132.1 (2)	N5—C10—H10	117.4
C7—N3—N2	105.8 (2)	N5—C11—H11A	109.5
C6—N4—C7	101.8 (2)	N5—C11—H11B	109.5
C10—N5—C11	122.1 (3)	N5—C11—H11C	109.5
C10—N5—C12	120.1 (3)	H11A—C11—H11B	109.5
C12—N5—C11	117.8 (3)	H11A—C11—H11C	109.5
N1—C1—H1	118.6	H11B—C11—H11C	109.5
N1—C1—C2	122.8 (3)	N5—C12—H12A	109.5
C2—C1—H1	118.6	N5—C12—H12B	109.5
C1—C2—H2	120.5	N5—C12—H12C	109.5
C1—C2—C3	118.9 (3)	H12A—C12—H12B	109.5
C3—C2—H2	120.5	H12A—C12—H12C	109.5
C2—C3—H3	120.5	H12B—C12—H12C	109.5
C4—C3—C2	119.0 (3)	O1—C13—C14	116.4 (3)
C4—C3—H3	120.5	O2—C13—O1	123.5 (3)
C3—C4—H4	120.6	O2—C13—C14	120.0 (3)
C3—C4—C5	118.9 (3)	C13—C14—H14A	109.5
C5—C4—H4	120.6	C13—C14—H14B	109.5
N1—C5—C4	122.1 (3)	C13—C14—H14C	109.5
N1—C5—C6	113.5 (2)	H14A—C14—H14B	109.5
C4—C5—C6	124.4 (3)	H14A—C14—H14C	109.5
N2—C6—C5	117.5 (3)	H14B—C14—H14C	109.5
			
Cu1—O1—C13—O2	−0.5 (4)	N3—C7—C8—C9	−118.2 (4)
Cu1—O1—C13—C14	178.2 (2)	N4—C7—C8—C9	59.2 (4)
Cu1—O3—C10—N5	172.0 (2)	C1—N1—C5—C4	1.8 (4)
Cu1^i^—N1—C1—C2	179.5 (2)	C1—N1—C5—C6	−178.5 (3)
Cu1^i^—N1—C5—C4	−178.8 (2)	C1—C2—C3—C4	1.0 (5)
Cu1^i^—N1—C5—C6	0.9 (3)	C2—C3—C4—C5	−0.4 (5)
Cu1^i^—N2—N3—Cu1	20.3 (4)	C3—C4—C5—N1	−1.0 (5)
Cu1^i^—N2—N3—C7	−158.4 (2)	C3—C4—C5—C6	179.3 (3)
Cu1^i^—N2—C6—N4	165.50 (19)	C4—C5—C6—N2	−171.7 (3)
Cu1^i^—N2—C6—C5	−13.5 (3)	C4—C5—C6—N4	9.4 (5)
Cu1—N3—C7—N4	−179.03 (19)	C5—N1—C1—C2	−1.2 (5)
Cu1—N3—C7—C8	−1.4 (5)	C6—N2—N3—Cu1	178.70 (18)
N1—C1—C2—C3	−0.2 (5)	C6—N2—N3—C7	0.1 (3)
N1—C5—C6—N2	8.5 (4)	C6—N4—C7—N3	0.8 (3)
N1—C5—C6—N4	−170.3 (3)	C6—N4—C7—C8	−176.8 (3)
N2—N3—C7—N4	−0.6 (3)	C7—N4—C6—N2	−0.8 (3)
N2—N3—C7—C8	177.1 (3)	C7—N4—C6—C5	178.1 (3)
N3—N2—C6—N4	0.5 (3)	C11—N5—C10—O3	−178.9 (3)
N3—N2—C6—C5	−178.5 (2)	C12—N5—C10—O3	1.0 (5)

Symmetry code: (i) −*x*+1, −*y*+1, −*z*+1.

### Hydrogen-bond geometry (Å, º)

**Table:**

*D*—H···*A*	*D*—H	H···*A*	*D*···*A*	*D*—H···*A*
C3—H3···O2^ii^	0.93	2.59	3.513 (4)	170

Symmetry code: (ii) −*x*+1, −*y*, −*z*+1.
